# Mental health characteristics associated with dysfunctional coronavirus anxiety

**DOI:** 10.1017/S003329172000121X

**Published:** 2020-04-16

**Authors:** Sherman A. Lee, Mary C. Jobe, Amanda A. Mathis

**Affiliations:** Department of Psychology, Christopher Newport University, Newport News, Virginia, USA

As of 2 April 2020, over one million people have been infected with a novel viral pneumonia (COVID-19) that emerged from Wuhan, China late December of 2019 (Johns Hopkins University, 2020). For most people, life has radically changed for the worse, as unprecedented rates of job loss, isolation, and COVID-19-related deaths and infections continue to soar. Although health professionals acknowledge the rising fear and anxiety of their patients and others around them, very little is known about those who are debilitated by their fear-based reactions to this infectious disease outbreak. There have been reports in China of elevated levels of depression, post-traumatic stress, anxiety, and insomnia among healthcare workers (Lai et al., 2020; Xiang et al., 2020) and their patients infected with COVID-19 (Bo et al., 2020), but the extent to which these psychological conditions are attributable to coronavirus anxiety has not been determined.

In a recent study of 775 adults residing in the U.S., individuals who were functionally impaired by their fear and anxiety of the coronavirus exhibited greater hopelessness, suicidal ideation, spiritual crisis, and alcohol/drug coping, than those who were anxious, but not impaired by the disease (Lee, in press). Because a large number of people tend to experience clinically significant fear and anxiety during an infectious disease outbreak (Taylor, 2019), it is vital for health professionals to understand the psychological challenges of those with this particular condition (Asmundson & Taylor, 2020). Thus, we examined online survey data from 1237 MTurk workers taken on 2 April 2020, in order to identify mental health characteristics of adults with dysfunctional coronavirus anxiety.

This sample consisted of 675 males, 558 females, 4 other gender, with a median age of 35 years (range 18−65). The rates of coronavirus infection, dysfunctional coronavirus anxiety (CAS ⩾ 9; Lee, in press), generalized anxiety (GAD-7 ⩾ 10; Spitzer, Kroenke, Williams, and Lowe, 2006), depression (PHQ ⩾ 10; Kroenke, Spitzer, and Williams, 2001), and functional impairment (WSAS ⩾ 21; Mundt, Marks, Shear, and Greist, 2002) were 4.9%, 25.4%, 36.0%, 40.3%, and 35.0%, respectively. A logistic regression, which controlled for sociodemographic effects of age, gender, education, and race, demonstrated that dysfunctional coronavirus anxiety was associated with coronavirus infection (odds ratio (OR) 3.04, 95% CI 1.28–7.25), generalized anxiety (OR 1.13, 95% CI 1.06–1.20), depression (OR 1.09, 95% CI 1.03–1.15), functional impairment (OR 1.08, 95% CI 1.05–1.11), perceived lack of social support (OR 1.16, 95% CI 1.04–1.28), and suicidal ideation (OR 1.24, 95% CI 1.13–1.37). This model explained 62% (Nagelkerke *R*^2^) of the variance in dysfunctional coronavirus anxiety and correctly classified 75% of cases.

These results suggest that people with dysfunctional coronavirus anxiety suffer from a wide range of psychological difficulties and that having the coronavirus infection poses a major risk factor for this form of psychopathology. These findings are part of a disturbing trend of coronavirus anxiety found among people in the U.S. (Lee, in press), China (Lai et al., 2020; Xiang et al., 2020), and more recently India, where a man committed suicide because he was afraid that he acquired the disease (Goyal, Chauhan, Chhikara, Gupta, & Singh, 2020). These findings also highlight the need for health professionals to screen for dysfunctional coronavirus anxiety, as it not only is linked to mental distress, but it may mimic, accompany, or be the underlying cause of other physical and mental complaints during this crisis (Taylor, 2019). Online mental health approaches, such as those implemented in China (Liu et al., 2020), may be particularly useful in evaluating and treating those with dysfunctional coronavirus anxiety.
